# Socio-demographic, clinical, and psychosocial factors associated with primary caregivers’ decisions regarding HIV disclosure to their child aged between 6 and 12 years living with HIV in Malawi

**DOI:** 10.1371/journal.pone.0210781

**Published:** 2019-01-15

**Authors:** Fatch Welcome Kalembo, Garth E. Kendall, Mohammed Ali, Angela F. Chimwaza

**Affiliations:** 1 School of Nursing, Midwifery and Paramedicine, Curtin University, Perth, Australia; 2 Faculty of Health Sciences, Mzuzu University, Mzuzu, Malawi; 3 Kamuzu College of Nursing, University of Malawi, Blantyre, Malawi; University of New South Wales, AUSTRALIA

## Abstract

The World Health Organisation (WHO) recommends that children living with HIV should be informed about their HIV status within the ages of 6 to 12 years using age-appropriate resources. The aim of this study was to assess the socio-demographic, clinical and psychosocial factors associated with primary caregivers’ decisions to disclose HIV to children living with HIV aged 6 to 12 years in Malawi. A cross-sectional study of 429 primary caregivers of children living with HIV were systematically recruited from all regions of the country. Information on HIV disclosure, family and child socio-demographic characteristics, child clinical characteristics, and child and family psychosocial characteristics was collected using validated instruments. Logistic regression was used to analyse data. The prevalence of non-disclosure of HIV status to children was 64 per cent. Concerns about the child’s inability to cope with the news (29%), a lack of knowledge on how to disclose HIV status (19%), and fear of stigma and discrimination (17%) were the main reasons for non-disclosure. On multivariate analysis, the odds of non-disclosure were higher among primary caregivers who were farmers (aOR 3.0; 95% CI: 1.1–8.4), in younger children (6–8 years) (aOR 4.1; 95% CI: 2.3–7.4), in children who were in WHO HIV clinical stage one (aOR 3.8; 95% CI: 1.4–10.2), and in children who were not asking why they were taking ARVs (aOR 2.9; 95% CI: 1.8–4.8). On the other hand, nondisclosure of HIV status was less likely in underweight children (aOR 0.6; 95% CI: 0.3–0.9). Many children living with HIV in Malawi are unaware of their HIV status. Non-disclosure is associated with a number of clinical and demographic characteristics. The findings highlight the need to provide guidance and support to primary caregivers to help them to effectively disclose HIV status to their children.

## Introduction

In 2015, the United Nations programme for HIV/AIDS (UNAIDS) estimated that one million people of all age groups, 6.7 per cent of the total population, were living with HIV in Malawi [1]. The preliminary results of the first national representative survey to measure the prevalence of HIV among children reported that 1.6 percent of children under the age of 15 years (84,000) were living with the virus in Malawi in 2016 [2]. This estimate is unlikely to be accurate due to the low survey response rate of 61.7% [2]. While the exact proportion of children living with HIV in Malawi has not yet been established, it is clear that HIV/AIDS has a significant impact. For example, in the same year, it was estimated that 530,000 children living in Malawi were orphaned due to the HIV-related death of both parents [1]. The magnitude of these numbers underscores the huge burden that HIV places on families and healthcare resources in Malawi.

The increase in coverage of anti-retroviral (ARV) medications among infected women in Malawi has led to a 71% decline in new infections among children from 16,000 cases in 2009 to 4800 in 2015 [1]. Moreover, with the widespread provision of ARVs at no direct cost to families, children with HIV are living longer than before [3]. Despite this improvement, Malawi and other sub-Saharan African countries fall well short of the survival rates now being achieved in high income countries such as the Netherlands and Australia [4–7]. In Australia, it is now expected that young adults living with HIV will have a normal lifespan if they take ARV medication routinely and lead a healthy lifestyle [6]. While it is important to acknowledge that children living with HIV in Malawi and other sub-Saharan countries face many challenges compared to their counterpart in high income countries due to the limitation of resources, many of them could also achieve a longer lifespan if they adhere to medication and receive adequate psychosocial support [8].

According to the American Academy of Paediatrics and the World Health Organisation (WHO), the early and progressive disclosure of HIV status to children aged between 6 and 12 years is critically important for their wellbeing [9, 10]. In 2011, following a systematic review of literature by an international group of academics and HIV experts, WHO published guidelines for HIV disclosure counselling for children up to 12 years of age [10]. The guidelines recommend that age-appropriate information be given to children as early as possible with full disclosure taking place by the time the child is 12 years of age [10].

Despite this recommendation, many children in Sub-Saharan Africa remain unaware of their HIV status [11]. There are many reasons why their HIV status is not disclosed. Studies in both resource-limited and resource-rich settings have pointed to the following factors as barriers to disclosure: HIV/AIDS-related stigma [12]; fear that the children might inappropriately disclose their HIV status to others, which would then lead to gossip, stigmatization, and discrimination towards them and their families [13]; concerns over the child’s reactions and potential psychological impact on the child’s health [14]; and caregivers’ lack of knowledge about the disclosure process [15].

Although Malawi has a large population of children living with HIV, there is limited evidence regarding the practice of HIV disclosure to children. In one of the few studies conducted in Malawi, it was revealed that parents were unwilling to have a conversation about HIV with their child because it was not culturally appropriate to talk to children about sexual issues [16]. In other studies, it has been found that parents were concerned that discussing HIV with their child might have a negative impact on the child’s wellbeing [16, 17]. Moreover, despite decades of positive HIV public health campaigns in Malawi, stigma, and discrimination directed at parents and children living with HIV are still common [17].

It is thus crucially important to estimate the current prevalence of HIV disclosure in Malawi and to identify socio-demographic and psychosocial characteristics of families and children associated with disclosure and non-disclosure. The aim of this study was to assess the sociodemographic, clinical, and psychosocial factors associated with primary caregiver’s decision to disclosure HIV to a child in Malawi.

## Methodology

### Study design and setting

This study was a cross-sectional study conducted in Malawi between March and June 2015. The study sites for primary caregivers were selected from the three administrative regions in Malawi, namely Northern, Central, and Southern regions. For administrative purposes, the country is further divided into 28 districts. Eight districts (three from the south, three from the centre and two from the north) were selected as study sites using a systematic approach. Districts were grouped under their respective Regions before assigning them numbers according to the number of districts in the Regions. Random numbers were then generated corresponding to the number of districts required in the region. The number of districts selected from each region as study sites was determined by the total number of districts in the region. In Malawi, each district has a district hospital which acts as a referral facility for all health centres and community hospitals within that district. In addition, each district hospital has an ART clinic. Normally children and their primary caregivers visit the clinics every two to three months to collect their ARV medication. Survey data were collected from primary caregivers at these ART clinics.

The sample size was calculated on the basis that the rate of HIV status disclosure to children in Malawi may be either high or low. In a recent study conducted in Kenya, Vreeman and colleagues [18] reported disclosure rates among children of 12 and 14 years of age of 44 per cent and 62 per cent, respectively. We used a sample size calculator developed by Rollin Bryant, https://www.stat.ubc.ca/~rollin/stats/ssize/b2.html. This sample size calculating formula was based on the estimation of sample size and power for comparing two binomial proportions in Bernard Rosner's Fundamentals of Biostatistics [19]. A sample size of 320 was required to provide 90 per cent power to detect a difference in the prevalence of HIV disclosure between different age groups, with a 5 per cent chance that a significant difference was due to chance [19]. Assuming a response rate of 75 per cent, approximately 427 potential participants were approached to participate in the survey.

### Participant recruitment and data collection procedure

Participants were eligible for the study if they met all of the following selection criteria: (a) the child was aged between 6 and 12 years and had been diagnosed with HIV and (b) the primary caregiver had cared for the child at least six months, was aged 18 years or older, had the ability to provide informed consent, and had not been diagnosed with a psychiatric illness. On the clinic day, study participants were recruited using a systematic approach. A member of the research team who was not part of the clinic staff gave the potential participants information related to aims, procedure, outcomes, benefits and risks of the study as well as their rights in relation to participation or withdraw from the study. Those who were willing to participate in the study were given consent forms to sign or put their thumbprint, if they could not read or write. The potential participants were also assured that their information will kept confidential, and that only members of the research team will have access to it. Potential participants were assigned an odd or even number starting from one based on the time of their arrival at the clinic. Those who were assigned an odd number were screened according to the inclusion/exclusion criteria prior to their participation in the study. We collected data of oldest child with HIV within the age range of 6–12 years if a primary caregiver had two or more children living with HIV in this age group.

The research team was assigned a room for data collection within each ART clinic. Research assistants with a background in nursing, public health, and clinical medicine who were not part of the clinic staff were recruited to assist with data collection. The research assistants were trained on data collection procedures prior to data collection. The normal waiting time for primary caregivers at the antiretroviral therapy clinic was two to three hours depending on the number of patients in attendance on that day. Data collection took place during the waiting time or after participants had been attended to by healthcare workers. To prevent inadvertent HIV status disclosure to children, they were separated from their primary caregivers during data collection and were entertained with cartoon shows on a portable DVD player in a separate room. One of the research assistants was assigned to look after the children while they were watching the cartoon. Face to face interviews were used to collect data from study participants. The first author, or one of the research assistants, asked the questions detailed in the questionnaire and recorded participants’ responses. The data collection process for each study participant took 30 to 40 minutes to be completed. The study participants were given a one-kilogram packet of sugar at the end of the interviews as a gift to compensate them for their time. The participants were not told about the gift until the interviews were completed so that it would not be an inducement to participate.

Ethics approvals were obtained from the Curtin University Human Research Ethics Committee and the Malawi Government National Health Science Research Committee prior to the commencement of data collection. Written informed consent was sought from the study participants prior to data collection.

### Data collection instruments

The questionnaire contained validated instruments to collect data about knowledge and practice of HIV disclosure, family socioeconomic and demographic characteristics, child demographic and clinical characteristics, child behavioural and emotional well-being [20], the burden of illness on the family [21], stressful life events [22], and family functional support [23]. The instruments were translated from English to Chichewa (Malawian local language) and then back-translated following established WHO guidelines for translation [24]. People of all ethnicities in Malawi speak and understand Chichewa. All instruments were piloted with primary caregivers of children living with HIV who were attending a health centre not included in the sample and corrected by members of the research team prior to the commencement of data collection.

### Primary caregiver knowledge and practice of HIV disclosure

Participants were asked questions regarding their knowledge, practice, barriers and facilitators of HIV disclosure to children (see S1 File).

### Family socio-demographic characteristics

The primary caregiver was asked questions relating to their socio-demographic status. Socio-economic status was assessed through the Wealth Index tool. The Wealth Index tool was developed by the World Bank to measure the socioeconomic status of people in developing countries using questions about the type of dwelling, availability of household goods, television, toilet facilities, the source of drinking water [25]. A composite index of economic status was created by applying weights from the 2010 Malawi National Demographic Survey to the collected data [26, 27]. The Wealth Index tool has been used previously and validated in Malawi [28, 29] (see S1 File).

### Child demographic and clinical characteristics

The primary caregiver was asked questions about the child including the child’s age, gender, child’s WHO HIV clinical stage (this information was obtained from the child’s health profile book), and child’s anthropometric measures (see S1 File). The 2000 Centre for Disease Control BMI for age percentile cut-offs were used in order to identify children who had either a normal weight, underweight, or overweight/obese [30].

### Child and family psychosocial characteristics

The Strengths and Difficulties Questionnaire (SDQ) was used to assess the behavioural and emotional wellbeing of children [20]. The SDQ is a widely used questionnaire which has been translated into many languages including Chichewa, the Malawian local language. The SDQ is a Likert scale with 25 items, each with three answer; options: not true, somewhat true, and certainly true. Although the instrument is not validated in Malawi, it has been widely used in studies in many African countries [31, 32]. The instrument has been reported adequate reliability (Cronbach’s alpha ranging from 0.78 to 0.82) and validity [20]. A binary variable (close to average and slightly raised/high/very high) was created from the newer band categorisation of the SDQ total difficulties score (0–13 ‘close to average’; 14–16 ‘slightly raised’; 17–19 ‘high’; > 19 ‘very high’) [33]. Close to average was classified as not having a behavioural or emotional problem while ‘slightly raised/high/very high’ was classified as having an emotional or behavioural problem [33].

The level of burden associated with caring for a child with HIV was assessed using the Impact on the Family Scale [21]. The scale has 24 items, each rated on a four-point Likert scale; strongly agree, agree, disagree and strongly disagree. Only 15 items were used to compute the total score following authors’ revised scoring instructions. The scale has acceptable internal reliability (Cronbach’s alpha ranging from 0.86 to 0.87) and validity as reported in a previous study conducted in the United States of America [21]. The total scores range from 15 to 60 with high scores indicating a great impact of child’s illness on the family and lower scores indicating little impact [34]. A level of impact variable with three categories (low-level impact, significant impact, and very serious impact) was then computed by applying the following cut-off scores; 30 or less as low-level impact, 31 to 45 as significant impact and above 45 as serious impact [34, 35].

Stressful life events experienced by the primary caregiver’s family were measured using the Life Stress Scale adapted from Tennant and Andrews [22]. The instrument had nine statements regarding stressful life events with yes and no option answers. Primary caregivers were asked to identify all those that they experienced in the last year. The scale has reported adequate reliability (Cronbach’s alpha 0.70 to 0.90) and validity in a study conducted in the United Kingdom [36]. A binary variable was created by classifying those who experienced less than three stressful life events in one category and those that experienced three or more stressful life events in another category [37].

The level of family support was assessed using the Support Function Scale [23]. The instrument has 20 items regarding different types of assistance that people sometimes find helpful. Each statement had five possible answers; never, once in a while, sometimes, often, and quite often. The scale has demonstrated adequate internal consistency (Cronbach’s alpha ranging from 0.77 to .87) validity in previous studies conducted in United States of America [23, 38]. Total scores range from 0 to 80 with high scores indicating less need for support and low scores indicating the great need for support. A binary categorical variable on level of support needed (high-level support or low-level support) was computed by applying cut-off scores of 55 or less for the great need for support and above 55 for less need for support [39].

### Statistical analysis

The main outcome of the study was HIV non-disclosure to children. The prevalence of child and family factors as well as the practice of HIV disclosure were tabulated. Bivariate analyses were conducted to assess the likelihood, or odds, of all child and family socioeconomic, demographic, clinical, and psychosocial variables being associated with HIV non-disclosure. Variables that were significantly associated with non-disclosure, with p-values of ≤ 0.25 in bivariate analysis [40] were entered into a multivariate logistic regression model in order to obtain the adjusted odds of these factors being associated with HIV non-disclosure. Data were analysed using SPSS version 22 and p-values were considered statistically significant at 0.05.

## Results

### Response rates

A total of 432 primary caregivers were approached to participate in the study. Three primary caregivers declined to participate; two were teachers on their way to school, while one needed to attend to family matters at home. A total of 429 primary caregivers were finally recruited into the study representing a response rate of 99.3 per cent.

### Family socio-demographic and child demographic and clinical characteristics

The prevalence of family sociodemographic and child characteristics are presented in Table 1. Seventy-seven per cent of participants were female, and of these, 61% were the biological mothers of children living with HIV. Half of the participants were 40 years of age or younger. Almost two thirds (64 percent) were married and 17 per cent were widowed. Slightly more than half (56%) had some primary education, while 22 per cent had no formal education. Forty-two per cent of caregivers were in the wealthiest category of the wealth index, while 10 per cent were poor, and 12 per cent were extremely poor. All the eight districts and three regions of Malawi were well represented. Mangochi, Nsanje, Kasungu, Karonga and Mzimba districts accounted for 13 per cent each of the total proportion of participants followed by Mulanje and Salima with 12 per cent each and Dowa had the least proportion (11%). In terms of regions, the Southern Region had the highest proportion of participants (38%), followed by the Central (37%) and Northern (25%). With regard to ethnicity, the Chewa accounted for 37 per cent of the primary caregivers followed by the Yao (17%), Lomwe and Sena had 13 per cent each, Tumbuka (12%) and other tribes (Tonga, Nkhonde, Mang’anja) 14 per cent.

**Table 1:** Family sociodemographic and child characteristics (N = 429).

Characteristic	n (%)	Characteristic	n (%)
Family sociodemographic characteristics		Child characteristics	
Relationship with the child		Age	
Mother	263 (61)	6–8	217 (51)
Father	63 (15)	9–10	100 (23)
Grandparent	50 (12)	11–12	112 (26)
Others*	53 (12)	Gender	
Age of primary caregiver		Male	221 (52)
18–30	51 (12)	Female	208 (48)
31–40	164 (38)	WHO HIV clinical staging	
41–50	132 (31)	Stage I	89 (21)
Above 50	82 (19)	Stage II	80 (19)
Gender of primary caregiver		Stage III	219 (51)
Male	99 (23)	Stage IV	41 (9)
Female	330 (77)	Nutritional status	
Marital status of primary caregiver		Underweight	258 (60)
Married	273 (64)	Normal	125 (29)
Single	43 (10)	Overweight/obese	46 (11)
Widowed	75 (17)	Nutritional status	
Divorced	38 (9)	Duration on ARVs ^a^ (n = 401)	
Education level of primary caregiver		≤ 1 year	78 (20)
None	94 (22)	2–3 years	129 (32)
Primary	240 (56)	≥4 years	194 (48)
Secondary/tertiary	95 (22)	Child asking why he/she is taking ARVs ^a^ (n = 401)	
Education level of spouse (n = 273)		Yes	204 (51)
None	47 (17)	No	197 (49)
Primary	134 (49)	Child refusing to take ARVs ^a^ (n = 401)	
Secondary/tertiary	92 (34)	Yes	291 (73)
No of children aged ≤12 years at home		No	110 (27)
≤ 2	312 (73)		
≥ 3	117 (27)		
No of children aged >12 years at home			
≤ 2	103 (24)		
≥ 3	326 (76)		
No of children aged >12 years at home			
≤ 2	103 (24)		
≥ 3	326 (76)		
Occupational status of primary caregiver			
Employed/self employed	131 (30)		
Farming	196 (46)		
Looking for a job	29 (7)		
Home duties	73 (17)		
Occupational status of spouse (n = 273)			
Employed/self employed	106 (39)		
Farming	110 (40)		
Looking for a Job	15 (6)		
Home duties	42 (15)		
Wealth quintiles			
Poorest	52 (12)		
Poor	43 (10)		
Middle	75 (18)		
Wealthy	78 (18)		
Wealthiest	181 (42)		

^a^Twenty-eight participants are missing in this variable because they were not yet on ARVs despite attending the ART clinic

*Uncle, aunt, sibling and legal guardian

In terms of the child characteristics, Table 1 shows that 51 per cent were between 6 to 8 years old, with the remaining older than 8 years of age. There were slightly more males (52%) than females (48%). Half of all children were at stage three of the WHO HIV clinical staging. More than half of the children (60%) were underweight.

### Prevalence of child and family psychosocial characteristics

Table 2 presents the prevalence of child and family psychosocial characteristics. Table 2 shows that 31 per cent of the children were identified as having an emotional or behavioural problem. Close to two-thirds (65%) of participants reported that the illness of the child had a significant or very serious impact on their families. About half of all primary caregivers (49%) reported that they had experienced three or more stressful life events in the past year. However, the great majority (80%) reported that they had a high level of social support.

**Table 2:** Prevalence of child and family psychosocial characteristics (N = 429).

Characteristic	n (%)
Child emotional and behavioural problems	
Close to average	296 (69)
Slightly high/high/very high	133 (31)
Level of impact of the child’s condition on the family	
Low level impact	106 (25)
Significant impact	252 (59)
Very serious impact	71 (16)
Level of functional support needed	
Low	344 (80)
High	85 (20)
Number of stressful life events	
<3	217 (51)
≥3	212 (49)

### Prevalence of primary caregiver knowledge and practice of HIV disclosure

Table 3 shows the prevalence of primary caregiver knowledge and practice of HIV disclosure. The prevalence of HIV non-disclosure was 64 per cent. Three-quarters of the children who knew their HIV status were told by their parents. The majority of children (60%) were first disclosed their HIV status when they were between 10 and 12 years of age. The reason for HIV disclosure varied among primary caregivers, with 24 per cent disclosing because they were advised to do so by healthcare workers, 23 per cent because the child was asking about his/her condition, and 21 per cent because they believed that the child was old enough to understand his or her HIV condition. On the other hand, the main reasons for non-disclosure were: concerns of the child’s inability to cope with the news (29%), a lack of knowledge on how to disclose HIV status (19%), and fear of stigma and discrimination (17%). Almost two-thirds of the participants (65%) identified the primary caregiver as the best person to disclose HIV status to a child, followed by the primary caregiver and healthcare worker disclosing together (20%), a healthcare worker (14%) and a teacher (1%).

**Table 3:** Prevalence of primary caregiver knowledge and practice of HIV disclosure (N = 429).

Characteristic	n (%)
Disclosure of HIV status	
Was the child disclosed his/her HIV status	
Yes	156 (36)
No	273 (64)
Who disclosed the HIV status to the child (n = 156)	
Parents	119 (76)
Healthcare worker	27 (17)
Grandparents	10 (7)
^c^Were HIV issues discussed prior to HIV disclosure (n = 156)	
Yes	109 (70)
No	47 (30)
How old was your child when his/her HIV status was first disclosed?	
Less than 6 years	3 (2)
6 years	7 (5)
7 years	12 (8)
8 years	17 (10)
9 years	19 (12)
10 years	28 (18)
11 years	31 (20)
12 years	34 (22)
I do not know	5 (3)
How was your child told about his/her HIV status (n = 156)	
As a one-time event	61 (39)
As a gradual process	95 (61)
*Reason for telling the child his/her HIV status (n = 156)	
Child is old enough to understand his condition	103 (26)
Advised by healthcare worker	92 (24)
Child asked about his illness	90 (23)
Child refusing to take HIV medicine	54 (14)
Child condition got worse	35 (9)
Parent condition got worse	17 (4)
Do you have adequate knowledge of HIV status disclosure	
Yes	149 (34)
No	280 (66)
Best person to disclose HIV to a child	
Primary caregiver	279 (65)
Healthcare worker	61 (14)
Teacher	2 (1)
Primary caregiver and healthcare worker	87 (20)
*Reasons for nondisclosure of HIV status to the child	
Child’s inability to handle the news	265 (29)
Fear of stigma and discrimination	153 (17)
Lack of support from healthcare workers	123 (13)
Lack of knowledge on how to disclose HIV status	172 (19)
Feelings of guilt or shame	116 (14)
The child not showing signs of sickness	86 (8)

^c^Health issues included causes, transmission and treatment

*Multiple response variable

### Association between family socio-demographic characteristics and non-disclosure of HIV status

Table 4 presents the association between family sociodemographic characteristics and non-disclosure to children living with HIV. In unadjusted bivariate analysis, primary caregivers who reported one or more of the following characteristics were more likely to report that their child was unaware of his or her HIV status: resident in Northern Malawi; having no more than two children older than 12 years; widowed; engaged in farming, and employed, or self-employed. The primary caregiver and spouses’ educational and the family socioeconomic status were not significantly associated with non-disclosure in bivariate analysis. In multivariate analysis, the odds of non-disclosure was higher among primary caregivers residing in Northern Malawi (aOR 2.7; 95% CI: 1.4–5.2); those who engaged in farming (aOR 3.0; 95% CI: 1.1–8.4); and who had no more than two children older than 12 years (aOR 3.8; 95% CI: 2.0–7.2).

**Table 4:** Factors associated with non-disclosure of HIV status in bivariate and multivariate analysis (N = 429).

Variable	Disclosed n (%)	Not disclosed n (%)	uOR (95% CI)	aOR (95% CI)
**Family socio-demographic factors**				
Region				
Central	71 (45)	88 (55)	1.0	1.0
South	56 (35)	105 (65)	1.5 (0.9–2.4)	1.5 (0.8–2.5)
North	29 (27)	80 (73)	2.2 (1.3–3.7)**	2.7 (1.4–5.2)**
Relationship with the child				
Father	24 (38)	39 (62)	1.0	
Other	20 (38)	33 (62)	1.0 (0.5–2.2)	
Grandparent	18 (36)	32 (64)	1.1 (0.5–2.4)	
Mother	94 (36)	169 (64)	1.1 (0.6–2.0)	
Age of primary caregiver				
18–30	20 (39)	31 (61)	1.0	
31–40	52 (32)	112 (68)	1.4 (0.7–2.7)	
41–50	52 (39)	80 (61)	1.0 (0.5–1.9)	
>50	32 (39)	50 (61)	1.0 (0.5–2.1)	
Gender of primary caregiver				
Male	36 (36)	63 (64)	1.0	
Female	120 (36)	210 (64)	1.0 (0.6–1.6)	
Number of children aged ≤12 years at home				
≤2	116 (37)	196 (63)	1.0	
≥3	40 (34)	77 (66)	1.1 (0.7–1.8)	
Number of children aged >12 years at home				
≤2	22 (21)	81 (79)	**2.6 (1.5–4.3)** ***	3.8 (2.0–7.2)***
≥3	134 (41)	192 (59)	1.0	
Marital status of primary caregiver				
Widowed	42 (56)	33 (44)	**0.3 (0.2–0.6)** ***	0.6 (0.3–1.1)
Single	18 (42)	25 (58)	0.6 (0.3–1.2)	0.5 (0.2–1.2)
Divorced	12 (32)	26 (68)	1.0 (0.5–2.0)	1.8 (0.8–4.2)
Married	84 (31)	189 (69)	1.0	1.0
Education level of primary caregiver				
None	36 (38)	58 (62)	1.0	
Primary	94 (39)	146 (61)	1.0 (0.6–1.6)	
Secondary/tertiary	26 (27)	69 (73)	1.6 (0.9–3.0)	
Education level of spouse^b^				
None	16 (34)	31 (66)	1.0	
Primary	42 (31)	92 (69)	1.1 (0.6–2.3)	
Secondary/tertiary	26 (28)	66 (72)	1.3 (0.6–2.8)	
Occupational status of primary caregiver				
Looking for a job	17 (59)	12 (41)	1.0	
Home duties	28 (38)	45 (62)	2.3 (0.9–5.5)	2.4 (0.8–7.3)
Farming	70 (36)	126 (64)	2.6 (1.2–5.6)*	3.0 (1.1–8.4)*
Employed/self employed	41 (31)	90 (69)	3.1 (1.4–7.1)**	2.3 **(**0.8–6.9)
^a^Occupational status of spouse				
Looking for a job	6 (40)	9 (60)	1.0	
Home duties	14 (33)	28 (67)	1.3 (0.4–4.5)	
Farming	35 (32)	75 (68)	1.4 (0.5–4.3)	
Employed/self employed	29 (27)	77 (73)	1.8 (0.6–5.4)	
Wealth quintiles				
Poorest	20 (39)	32 (61)	1.0	
Poor	15 (35)	28 (65)	1.2 (0.5–2.7)	
Medium	30 (40)	45 (60)	0.9 (0.5–1.9)	
Wealthy	38 (49)	40 (51)	0.7 (0.3–1.3)	
Wealthiest	53 (29)	128 (71)	1.5 (0.8–2.9)	
**Child demographic and clinical factors**				
Child’s age				
6–8	44 (20)	173 (80)	5.4 (3.3–9.0)***	4.1 (2.3–7.4)***
9–10	47 (47)	53 (53)	1.6 (0.9–2.7)	1.7 (0.8–3.1)
11–12	65 (58)	47 (42)	1.0	1.0
Child’s gender				
Female	78 (38)	130 (62)	1.0	
Male	78 (35)	143 (65)	1.1 (0.7–1.6)	
WHO HIV clinical staging				
Stage I	20 (22)	69 (78)	3.3 (1.5–7.2)**	**3.8 (1.4–10.2)** **
Stage II	32 (40)	48 (60)	1.4 (0.7–3.1)	1.6 (0.6–4.1)
Stage III	84 (28)	135 (62)	1.5 (0.8–3.0)	2.4 (0.9–5.6)
Stage IV	20 (49)	21 (51)	1.0	1.0
Nutritional status				
Normal	35 (28)	90 (72)	1.0	1.0
Underweight	109 (42)	149 (58)	0.5 (0.3–0.8)*	0.6 (0.3–0.9)*
Overweight/obese	12 (26)	34 (74)	1.1 (0.5–2.4)	0.8 (0.3–2.0)
^b^Duration on ARVs				
≤ 1 year	25 (32)	53 (68)	1.6 (0.9–2.8)	
2–3 years	44 (34)	85 (66)	1.5 (0.9–2.3)	
≥4 years	84 (43)	110 (57)	1.0	
^b^Child asking why he/she is taking ARVs				
Yes	97 (49)	100 (51)	1.0	1.0
No	56 (27)	148 (73)	2.6 (1.7–3.9)***	2.9 (1.7–4.7)***
^b^Child refusing to take ARVs				
Yes	44 (40)	66 (60)	1.0	
No	109 (38)	182 (63)	1.1 (0.7–1.7)	
**Child and family psychosocial factors**				
Level of impact of the child condition on family				
Low level impact	37 (35)	69 (65)	1.3 (0.7–2.5)	
Significant impact	90 (36)	162 (64)	1.2 (0.7–2.1)	
Very serious impact	29 (41)	42 (59)	1.0	
Level of functional support needed				
Low	125 (36)	219 (64)	1.0	
High	31 (36)	54 (64)	1.0 (0.6–1.7)	
Number of stressful life events				
<3	74 (34)	143 (66)	1.3 (0.9–1.9)	
≥3	82 (39)	130 (61)	1.0	
Scores of difficulties scale				
Close to average	125 (36)	220 (64)	1.0 (0.7–1.6)	
Slightly high/high/very high	31 (37)	53 (63)	1.0	

***P<0.001

**p<0.01

*p<0.05; Adjusted for variables in the table

^a^ 156 participants are missing in this variable because they had no spouse

^b^Twenty-eight participants are missing in this variable because they were not yet on ARVs despite attending the ART clinic

uOR- unadjusted odds ratio, aOR-adjusted odds ratio, ARVs- antiretrovirals

### Association between child demographic and clinical factors and non-disclosure of HIV status

Table 4 shows the association between child demographic and clinical factors and non-disclosure of HIV status. Child characteristics that significantly predicted non-disclosure of HIV status in multivariate statistics included: being 6–8 years of age (aOR 4.1; 95% CI: 2.3–7.4); WHO HIV clinical stage one (aOR3.8; 95% CI: 1.4–10.2), of underweight (aOR 0.6; 95% CI: 0.3–0.9) and children not asking why they were taking ARVs (aOR 2.9; 95% CI: 1.8–4.8).

### Association between child and family psychosocial factors and non-disclosure of HIV status

The association between child and family psychosocial factors and non-disclosure of HIV status are presented in Table 4. In bivariate analysis, the odds of non-disclosure were higher among primary caregivers who reported a low level of impact of illness (uOR 1.3; 95% CI: 0.7–2.5) and a significant level of impact of illness (uOR 1.2; 95% CI: 0.7–2.1) on the family compared to those who reported very serious level of impact. However, the associations were statistically insignificant. In addition, non-disclosure of HIV status was also not significantly associated with the need for functional support and experience of stressful life events.

## Discussion

In this study, the prevalence of non-disclosure among six to 12-year-old Malawian children living with HIV was 64%. The main reasons for non-disclosure were concerns about the child’s ability to cope with the news about his/her HIV status, lack of knowledge on disclosure, and fear of stigma and discrimination. Primary caregivers who were engaged in farming, resident in Northern Malawi, or living with two or fewer children older than 12 years had a higher likelihood of non-disclosure. In addition, non-disclosure of HIV status was more likely for children who were younger, in stage one of the WHO HIV clinical stage, and those who were not asking why they were taking ARVs. On the other hand, non-disclosure was less likely in children who were underweight. Child/family psychosocial factors, family wealth and parents' level of education were not associated with non-disclosure.

The prevalence of HIV non-disclosure in this study (64%) is similar to those reported in studies conducted in Tanzania [41], Zambia [31], South Africa [42], and Nigeria [43], (range 63–67%) and lower than those reported in two studies conducted in Kenya where the non-disclosure rates were 89 per cent [44] and 74 per cent respectively [18] and an Ethiopian study where the rate was 83 per cent [45]. While both the current study and the Kenyan study conducted by Turissini and colleagues (2013b) were cross-sectional and targeted children in a similar age group, data for the Kenyan study were collected from only one hospital, thus may not have been representative of all hospitals in Kenya. While our study used data from primary caregivers to determine the rate of non-disclosure, Vreeman and colleagues (2014) in Kenya, and Biadgilign and colleagues (2011) in Ethiopia used data collected from caregiver-child dyads to determine the rate of non-disclosure. Two studies conducted in sub-Saharan Africa found that caregivers tend to underreport non-disclosure compared to children [45, 46].

On the other hand, the prevalence of non-disclosure in our study is higher than those reported in two studies conducted in Uganda, where the non-disclosure rate was found to be 49 per cent [47] and 44 per cent [46]. As with the study conducted by Turissini and colleagues (2013) in Kenya, data for the Ugandan studies were collected at a limited number of sites and thus may not have been representative of the population. Furthermore, the focus of the two Ugandan studies was disclosure among children up to 18 years of age. Our finding that Malawian children aged 9 to 12 years were more likely than their younger counterparts to be aware of their HIV diagnosis supports this evidence that older children are more likely than younger children to be told that they have HIV [18, 48].

There is evidence that primary caregivers perceive younger children to lack the emotional and cognitive maturity to cope with knowledge of their HIV status [49]. Primary caregivers in this study also reported that they disclosed when the child was asking questions about taking HIV medication. These findings are consistent with those of a study conducted in Rwanda, where children who engaged in conversation with their primary caregivers about their condition were 15 times more likely to be told about their HIV status compared to those who were not [50]. Older children are simply more likely to ask questions and engage adults in conversation. For example, Madiba (2016) found that caregivers in Botswana and South Africa interpreted children’s frequent questions about their condition as a sign of maturity, that they are ready to be told about their HIV status [51].

Conversely, three recent studies conducted in Burkina Faso, Uganda, and The Democratic Republic of Congo found that despite children’s curiosity to know about their disease, primary caregivers felt that they were too young to be engaged in conversation about their HIV status and opted for deception and threats as a way of protecting the children from the perceived negative consequences of disclosure [12, 52–54]. There is a great deal of evidence that delay in telling children about their HIV status has negative implications for future health and wellbeing. For example, poor medication adherence can result in low virological suppression and the virus becoming drug resistant [55], the child unknowingly transmitting the virus to others [56], and failure of the child to assume independence and responsibility in HIV treatment and care [56].

Our finding that primary caregiver’s farming occupation was independently associated with non-disclosure is in contrast to the findings of two recent studies conducted in Tanzania and Rwanda, where farming occupation of parents was not significantly associated with non-disclosure of HIV status to children [41, 50]. The main source of income for people in districts where data were collected is farming [57]. Many of these farmers spend most of the day time in the field and may not have time or may be tired to sit down and talk about HIV disclosure with their children.

In addition, our study found that participants from the Northern Region of Malawi were more likely to have children who were unaware of their HIV status compared to their Central Region counterparts. This difference in non-disclosure may be due to the provision of comprehensive care to children living with HIV by Baylor College of Medicine Children’s Foundation in several districts in the Central Region. Baylor College of Medicine Children’s Foundation, which is a privately funded medical facility, only support HIV paediatric care in Government hospitals in the Central Region. In contrast, the Government hospitals in the Northern Region rely on Government for funding and have fewer resources to effectively promote disclosure of HIV status to children.

Furthermore, we found that living with less than three children older than 12 years was associated with non-disclosure. In Malawi, as in South Africa, older children are becoming a source of social support to their siblings and primary caregivers in families affected by HIV [58]. Since having older siblings in the family may increase support for the child living with HIV, the primary caregiver may disclose to the child with the expectation that this additional support will be available. Finally, we found that children who were in WHO HIV clinical stage one were more likely to be unaware of their HIV status compared to those in stage four. On the other hand, non-disclosure was less likely among children were underweight compared to those with normal weight. We can find no explanation for the relationship between weight and disclosure beyond speculation. A similar study conducted in Kenya reported no association between malnutrition and HIV disclosure to children [18]. With regard to the relationship between severity of symptoms and disclosure, previous findings are mixed [59–62].

Our study is one of the few in sub-Saharan Africa where the association between psychosocial factors and non-disclosure of HIV status to children was assessed [31, 63]. While we did not find any significant association between emotional and behavioural problems and non-disclosure, studies conducted in Kenya and Zambia have reported higher rates of mental health problems among children who were unaware of their HIV status [31, 63]. The younger age range of the children in the current study may account for this discrepancy.

The main reasons for non-disclosure reported in this study concur with accounts in other studies conducted in sub-Saharan Africa [49, 55, 64, 65]. While many primary caregivers are concerned that HIV disclosure will cause their child to experience psychological distress, the literature reveals that most children with HIV understand the seriousness of the condition long before he or she is told [66, 67]. Moreover, although many parents are worried that children might have emotional and behavioural difficulties when they are told about their HIV status, research has shown that these are usually short lived and disappear with time [55]. In addition, authors of a recent qualitative study in Uganda reported that primary caregivers were reluctant to disclose HIV status to children for fear of damaging their relationship with their children, nonetheless, children who knew their HIV status had a short lived anger towards their primary caregivers but they expressed concern for their primary caregiver’s delay in disclosing HIV status to them [68].

With regard to HIV stigma and discrimination, three qualitative studies conducted in Malawi show that these are still major problems despite the increased coverage of health promotion campaigns against such practices [16, 17, 69]. According to Pindani and colleagues (2014), people in Malawi living with HIV are discriminated against because many people believe that those with HIV: have been involved in socially unacceptable practices, such as sex work; are not moral; are infectious; and are incurable [69]. Furthermore, it has been found that stigma and discrimination are directed at all members of families that are affected by HIV, including children [16].

Primary caregivers’ lack of confidence in their ability to disclose appropriately is another major barrier to HIV disclosure that has been reported in previous studies [70–72]. These findings highlight the crucial need for healthcare workers to support primary caregivers appropriately through the disclosure process. It is difficult for primary caregivers to implement the WHO recommendation about gradual disclosure in an age-appropriate manner without a great deal of support from healthcare workers [10, 73, 74]. For example, authors of a qualitative study in Nigeria reported that parents did not know how to disclose HIV to their children and they asked for support from healthcare workers [75]. Within the context of a trusting relationship, it is essential for primary caregivers to understand why it is important to disclose and to develop the skills necessary to do this in a safe and effective manner.

### Strengths and limitations of the study

This study has a number of strengths. First, the study had a fairly representative data collected from the three regions of Malawi. Nonetheless, it may have biases considering that it excluded primary caregivers whose wards were receiving care at lower-level health centres and private hospitals. Primary caregivers of children receiving care in health centres were excluded because the centres do not have specialised HIV clinics and are situated in remote localities. Primary caregivers in private hospitals were excluded because only a small proportion of children living with HIV receive care in private hospitals. Second, this study assessed disclosure of HIV to children younger than 13 years as such it provides a good benchmark for evaluation of the implementation of the WHO guideline for HIV disclosure [10]. Notwithstanding the robust methodology, this study has limitations. The cross-sectional design of the study has limitations in making causal relationships about factors associated with non-disclosure of HIV status. In addition, although disclosure of HIV status is a gradual process, this study did not report the prevalence of children who had incomplete knowledge of their HIV status (partial disclosure). Future studies should include assessment of partial disclosure in order to have a more complete understanding of the disclosure process among children living with HIV in Malawi. Another limitation of the study is that some of the instruments used in this study such as Impact on the Family Scale, Support Functional Scale and Life Stress Scale used in this study were not validated in Malawi or other African countries. Nonetheless, these instruments were reviewed by language and academic experts in Malawi and piloted before commencement of data collection. In addition, we did not assess caregiver’s HIV status despite being an important factor in disclosure of HIV status to children in previous studies. We felt that it was not necessary to ask primary caregivers about their own HIV status because there is ample evidence that the great majority of children who have HIV in sub-Saharan Africa contract the disease through perinatal mother-to-child transmission [76–78].

## Conclusion

The prevalence of non-disclosure in Malawi is high. It is clear that primary caregivers in all socioeconomic and demographic groups struggle with the task. There are many reasons why caregivers choose not to disclose, especially to younger children. The results of this study indicate that providing age appropriate disclosure is a complex process for primary caregivers. There is some evidence from previous studies that healthcare workers can make this task easier for primary caregivers by providing appropriate guidance and support [73, 74]. We believe there is great potential for interventions to be developed to support both primary caregivers and healthcare workers in the disclosure process. Future research is warranted, including both longitudinal studies to better understand the disclosure process that takes place over time and intervention studies that begin by asking primary caregivers and healthcare workers to identify the kinds of resources they require to help them effectively disclose.

## Supporting information

S1 File Questionnaire. (DOCX)
