# Supplementary material for: Socio-demographic, clinical, and psychosocial factors associated with primary caregivers’ decisions regarding HIV disclosure to their child aged between 6 and 12 years living with HIV in Malawi
Source: PLoS One. 2019 Jan 15;14(1):e0210781. doi: 10.1371/journal.pone.0210781 (PMC6333381; doi:10.1371/journal.pone.0210781)
Supplement: S1 File — (DOCX) [file pone.0210781.s001.docx]

**S 1 File. Questionnaire**


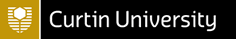


**Study title: Psychosocial and health system factors in disclosure of HIV status to children living with HIV in Malawi: Needs assessment and formative evaluation of a disclosure resource.**

The purpose of this questionnaire is to obtain information about you, your partner, your child, household, finances, occupation, and disclosure of HIV status to the child, need for development of HIV status disclosure resource, child’s physical and emotional health, life stress, social support and impact of the child’s illness on the family.

The questionnaire will take 30 to 45 minutes to complete. Please respond to all questions with honesty, completeness and accuracy. You are free to withdraw from participating in this study at any time without penalty. All answers you give will be confidential. Please use the pen to complete the questionnaire.

Tick the box that corresponds to your answer and write clearly where required. For example: How old is your child

6 years ☒ 1

7 years ☐ 2

8 years ☐ 3

9 years ☐ 4

10 years ☐ 5

11years ☐ 6

12years ☐ 7

If your answer is 6 years, then tick the box next to 6 years with a pen as indicated above.

| **PART A: SOCIO-DEMOGRAPHIC DATA.** | | |
| --- | --- | --- |
|  | | |
| A1. | What is your relationship with the child? | |
|  | Mother ☐ | Uncle ☐ |
|  | Father ☐ | Grandparent ☐ |
|  | Aunt ☐ | Sibling ☐ |
|  |  | Legal guardian ☐ |
|  | Other (please specify)… | |
|  | | |
| A2. | What is your age? | |
|  | Below 15 years ☐ | 36-40 years ☐ |
|  | 15- 20 years ☐ | 41-45 years ☐ |
|  | 21-25 years ☐ | 46-50 years ☐ |
|  | 26-30 years ☐ | Over 50 years ☐ |
|  | 31-35 years ☐ |  |
|  | | |
| A3. | What is your gender? |  |
|  | Male ☐ | Female ☐ |
|  | | |
| A4. | What is your current marital status? | |
|  | Married ☐ | Widowed ☐ |
|  | Single ☐ | Divorced ☐ |
|  | | |
| A5. | What is the highest level of formal education you have completed? | |
|  | None ☐ | College ☐ |
|  | Primary ☐ | University ☐ |
|  | Secondary ☐ |  |
|  | | |
| A6. | What is the highest level of formal education your spouse has completed? | |
|  | None ☐ | College ☐ |
|  | Primary ☐ | University ☐ |
|  | Secondary ☐ | Not applicable ☐ |
|  | | |
| A7. | How many children 12 years of age or younger live in your home? | |
|  | 1-2 ☐ | 6-8 ☐ |
|  | 3-5 ☐ | 9-12 ☐ |
|  | | |
| A8. | How many children over 12 years of age live in your home? | |
|  | 0 ☐ | 6-8 ☐ |
|  | 1-2 ☐ | 9-12 ☐ |
|  | 3-5 ☐ |  |
|  | | |
| A9 | Which of the following would you describe your tribe as? | |
|  | Chewa ☐ | Ngoni ☐ |
|  | Yao ☐ | Tonga ☐ |
|  | Tumbuka ☐ | Sena ☐ |
|  | Lomwe ☐ | Nkhonde ☐ |
|  | Other (please specify)…………………………………………………………… |  |

| A10. | Does your household have any of the following (tick all that apply) ? | | |
| --- | --- | --- | --- |
|  | Electricity | Yes ☐ | No ☐ |
|  | Koloboyi | Yes ☐ | No ☐ |
|  | A paraffin lamp other than a koloboyi | Yes ☐ | No ☐ |
|  | A radio? | Yes ☐ | No ☐ |
|  | A television | Yes ☐ | No ☐ |
|  | A cellular phone | Yes ☐ | No ☐ |
|  | A telephone (landline) | Yes ☐ | No ☐ |
|  | A bed with a mattress | Yes ☐ | No ☐ |
|  | A sofa set? | Yes ☐ | No ☐ |
|  | A table and chair(s) | Yes ☐ | No ☐ |
|  | A refrigerator? | Yes ☐ | No ☐ |
|  | A bicycle | Yes ☐ | No ☐ |
|  | Motorcycle or scooter | Yes ☐ | No ☐ |
|  | An oxcart | Yes ☐ | No ☐ |
|  | A car | Yes ☐ | No ☐ |
|  | | | |
| A11. | What is the main source of drinking water for members of your household?(Tick only one) | | |
|  | Piped water | Water from spring | |
|  | Piped into dwelling ☐ | Protected spring ☐ | |
|  | Piped to yard or plot ☐ | Unprotected spring ☐ | |
|  | Public taps/standpipe ☐ | Rain water ☐ | |
|  | Borehole ☐ | Surface water ☐ | |
|  | Dug well | Bottled water ☐ | |
|  | Protected well ☐ |  | |
|  | Unprotected well ☐ |  | |
|  | Other (please specify….……...…………………………………………………………………….. | | |
|  | | | |
| A12. | What kind of toilet facility is used by members of your household? | | |
|  | Flush toilet ☐ | Compositing toilet ☐ | |
|  | Ventilated improved latrine ☐ | Bucket toilet ☐ | |
|  | Pit latrine with a slab ☐ | No facility/bush/field ☐ | |
|  | Pit latrine without a slab/one pit ☐ |  | |
|  | | | |
| A13. | What type of fuel is used for heating by your household? | | |
|  | Electricity ☐ | Wood ☐ | |
|  | Natural gas ☐ | Straw/shrubs/grass ☐ | |
|  | Kerosene ☐ | Animal dung ☐ | |
|  | Coal ☐ | No food cooked in household ☐ | |
|  | Charcoal ☐ |  | |
|  | Other (please specify……………………………………………………………………………………… | | |

| A14. | What is the main material of the floor of your house | |
| --- | --- | --- |
|  | Earth/sand ☐ | Broken bricks ☐ |
|  | Dung ☐ | Ceramic tiles ☐ |
|  | Wooden planks ☐ | Cement ☐ |
|  | Palm/Bamboo/grass ☐ |  |
|  | Other (please specify)………………………………………………………………………………….. | |

| A15. | What is the main material of the roof of your house? | |
| --- | --- | --- |
|  | No roof ☐ | Iron sheets ☐ |
|  | Thatch/palm leaf ☐ | Wood ☐ |
|  | Palm/bamboo/grass ☐ | Cement ☐ |
|  | Wood planks ☐ | Ceramic tiles ☐ |
|  | Cardboard ☐ |  |
|  | Other (Please specify………………………………………………………………………………........... | |
|  | | |
| A16. | What is the main material of the exterior walls of your house? | |
|  | No walls ☐ | Cement ☐ |
|  | Cane/palm/trunks ☐ | Stone with lime/cement ☐ |
|  | Bamboo/tree trunks with mud ☐ | Burnt bricks ☐ |
|  | Stone with mud ☐ | Unburnt bricks ☐ |
|  | Plywood ☐ | Cement blocks ☐ |
|  | Cardboard ☐ | Wood planks ☐ |
|  | Other (please specify) ……………………………………………………………………………… | |
|  | | |
| A17. | Does your household own any livestock, herds, other farm animals, or poultry? | |
|  | Yes ☐ | No ☐ Go to A19 |
|  | | |
| A18. | How many of the following animals does your household own? (Specify number) | |
|  | Goats...................... | Chickens…………… |
|  | Pigs......................... | Ducks……………… |
|  | Cattle...................... | Pigeons…………… |
|  | Other (please specify) ……………………………………………………………………………..... | |
|  | | |
| A19. | Does any member of your household own any agricultural land? | |
|  | Yes ☐ | No ☐ Go to A21 |
|  | | |
| A20. | How much agricultural land do members of your household own? (Specify number) | |
|  | Acres …………………. | Football pitches……….. |
|  | Hectares………………. | I don’t know ☐ |
|  |  |  |
| A21. | In the last 7 days, how many main meals did your household have per day? | |
|  | 1 ☐ | 3 or more ☐ |
|  | 2 ☐ |  |
|  | | |
| A22. | In the last year, did your household have sufficient food? | |
|  | Yes ☐ | No ☐ |

| A23. | What do you currently spend most of your time doing? (Please mark only one answer, unless two, or more, answers apply equally) | |
| --- | --- | --- |
|  | Full-time or part-time job (salary or own business) ☐ | Recovering from injury / illness ☐ |
|  | Farming ☐ | Caring for an ill person ☐ |
|  | Looking for work ☐ | Studying ☐ |
|  | Home duties / caring for children ☐ |  |
|  |  |  |
| A24. | What does your spouse currently spend most of his/her time doing? (Please mark only one answer, unless two, or more, answers apply equally) | |
|  | Full-time or part-time job (salary or own business) ☐ | Recovering from injury / illness ☐ |
|  | Farming ☐ | Caring for an ill person ☐ |
|  | Looking for work ☐ | Studying ☐ |
|  | Home duties / caring for children ☐ | Not applicable ☐ |
|  | | |
| **PART B: ABOUT YOUR CHILD OR THE CHILD YOU ARE CARING FOR. THIS SECTION, ASKS ABOUT YOUR OLDEST CHILD WITH HIV WITHIN THE AGE RANGE OF 6-12 YEARS.** | | |
|  | | |
| B1. | What is your child’s age? | |
|  | 6 years ☐ | 10 years ☐ |
|  | 7 years ☐ | 11 years ☐ |
|  | 8 years ☐ | 12 years ☐ |
|  | 9 years ☐ |  |
|  | | |
| B2. | What is your child’s gender? |  |
|  | Female ☐ | Male ☐ |

| B3. | What is your child’s WHO clinical staging of HIV disease? (Obtain from the child’s health profile book) | | |
| --- | --- | --- | --- |
|  | Stage 1 ☐ | Stage 3 ☐ | |
|  | Stage 2 ☐ | Stage 4 ☐ | |
|  |  |  |  |
| B4. | What are your child’s anthropometric measures? (Please measure) | | |
|  | Height …………………(M) | Weight.……………(Kg) | |
|  |  |  | |
| B5. | Does your child have now, or has your child had in the last year, any of the following health professional diagnosed medical conditions or health problems? (Please tick all that apply) | | |
|  | Fever | Yes ☐ No ☐ | |
|  | Diarrhoea | Yes ☐ No ☐ | |
|  | Pneumonia | Yes ☐ No ☐ | |
|  | Tuberculosis | Yes ☐ No ☐ | |
|  | Skin disease | Yes ☐ No ☐ | |
|  | Undernutrition | Yes ☐ No ☐ | |
|  | Other (please specify) ……………………………………………………………………………… | | |
|  | | | |
| B6. | Is your child currently taking HIV medicine? | | |
|  | No ☐ | Yes ☐ | |

| B7. | | If yes, for how long has your child been on the HIV medicine? | | | |
| --- | --- | --- | --- | --- | --- |
|  | | Less than 6 months ☐ | | 4 to 5 years ☐ | |
|  | | 6 months to 1 year ☐ | | More than 5 years ☐ | |
|  | | 2 to 3 years ☐ | |  | |
|  |  |  |  |  |  |
| **PART C. DISCLOSURE OF HIV STATUS TO YOUR CHILD** | | | | | |
| C1. | | Does your child know that he/she has HIV? | | | |
|  | | Yes ☐ | | No ☐ (Go to C7) | |
|  | | | | | |
| C2. | | Who disclosed the HIV status to your child? (Tick all that apply) | | | |
|  | | Parents ☐ | | Aunt ☐ | |
|  | | Healthcare worker ☐ | | Grandparent ☐ | |
|  | | Uncle ☐ | | Legal guardian ☐ | |
|  | | Other (please specify) ……………………………………………………………………………… | | | |
| C3. | | Were HIV related issues such as causes, transmission and treatment, discussed with the child prior to disclosure of HIV status? | | | |
|  | | Yes ☐ | | Not sure ☐ | |
|  | | No ☐ | |  | |
|  | | | | | |
| C4 | | How was your child told about his/her HIV status? | | | |
|  | | As a one-time event ☐ | | Not sure ☐ | |
|  | | As a gradual process ☐ | |  | |
|  | | | | | |
| C5. | | How old was your child when his/her HIV status was first disclosed? | | | |
|  | | Less than 6 years ☐ | | 9 years ☐ | |
|  | | 6 years ☐ | | 10 years ☐ | |
|  | | 7 years ☐ | | 11 years ☐ | |
|  | | 8 years ☐ | | 12 years ☐ | |
|  | | | | | |
| C6. | | Which of the following statements best describes the reason for telling your child his HIV status? (Tick all that apply) | | | |
|  | | The child is old enough to understand his condition | Yes ☐ | | No ☐ |
|  | | The healthcare worker insisted that the child should be told of his/her condition | Yes ☐ | | No ☐ |
|  | | The child asked about his illness | Yes ☐ | | No ☐ |
|  | | The child did not want to take medicines because he/she did not know why he/she was taking them | Yes ☐ | | No ☐ |
|  | | The child’s condition got worse | Yes ☐ | | No ☐ |
|  | | The parent’s health condition got worse | Yes ☐ | | No ☐ |
|  | | Other (please specify)……………………………………………………………………………………… | | | |

| C7. | Please answer the following general questions about HIV disclosure | | | | |
| --- | --- | --- | --- | --- | --- |
|  | Does the child know that he /she is taking medicines for HIV? | Yes ☐ | | No ☐ | Not sure ☐ |
|  | Does the child ever ask questions about why he /she is taking medicine? | Yes ☐ | | No ☐ | Not sure ☐ |
|  | Does the child ever refuse to take medicines that he or she is supposed to take? | Yes ☐ | | No ☐ | Not sure ☐ |
|  | Do you ever not give medicines to the child because you do not want to give them in front of other people? | Yes ☐ | | No ☐ | Not sure ☐ |
|  | Do you ever have problems with giving the medicines because the child does not know why he/she taking them? | Yes ☐ | | No ☐ | Not sure ☐ |
|  | Does the child ever have problems taking the medicines on time or taking them every day? | Yes ☐ | | No ☐ | Not sure ☐ |
|  | Do other children avoid playing with the child because of his/her HIV status? | Yes ☐ | | No ☐ | Not sure ☐ |
|  | Do other children tease or call the child hurtful names because of his/her HIV status? | Yes ☐ | | No ☐ | Not sure ☐ |
|  | Has your child been rejected by friends or family because of his illness? | Yes ☐ | | No ☐ | Not sure ☐ |
|  | Does your child seem to have little pleasure in doing things lately? | Yes ☐ | | No ☐ | Not sure ☐ |
|  | Has your child been feeling down, depressed or hopeless? | Yes ☐ | | No ☐ | Not sure ☐ |
|  | | | | | |
| C8. | Who do you think is the best person to disclose HIV status to children living with HIV? | | | | |
|  | Primary caregiver ☐ | | Healthcare worker ☐ | | |
|  | Teacher ☐ | | Church minister ☐ | | |
|  | Other (please specify) ……………………………………………………………………………… | | | | |
|  | | | | | |
| C9. | Do you think you have adequate knowledge on how to disclose HIV status to your child? | | | | |
|  | Yes ☐ | | Not sure ☐ | | |
|  | No ☐ | |  | | |

| C10. | Which of the following statement best describes the reason that prevent you from telling your child about his/her HIV status? (Tick all that apply) (If disclosed go to Question C11) | | |
| --- | --- | --- | --- |
|  | Fear of children’s inability to handle the news (mental immaturity) | Yes ☐ | No ☐ |
|  | Fear of stigma and discrimination | Yes ☐ | No ☐ |
|  | Lack of support from the healthcare workers | Yes ☐ | No ☐ |
|  | Lack of knowledge on how to disclose | Yes ☐ | No ☐ |
|  | Feeling of guilty or shame | Yes ☐ | No ☐ |
|  | Because the child is not showing signs of sickness | Yes ☐ | No ☐ |
|  | Other (please specify)……………………………………………………………………………………… | | |

**PART D. CHILD DEVELOPMENT**

| D1. For each item, please mark the box for Not True, Somewhat True or Certainly True. Please give your answers on the basis of your child's behaviour over the last six months | | | |
| --- | --- | --- | --- |
|  | Not true | Somewhat True | Certainly True |
| Considerate of other people's feelings | ○ | ○ | ○ |
| Restless, overactive, cannot stay still for long | ○ | ○ | ○ |
| Often complains of headaches, stomach-aches or sickness | ○ | ○ | ○ |
| Shares readily with other children (treats, toys, pencils etc.) | ○ | ○ | ○ |
| Often has temper tantrums or hot tempers | ○ | ○ | ○ |
| Rather solitary, tends to play alone | ○ | ○ | ○ |
| Generally obedient, usually does what adults request | ○ | ○ | ○ |
| Many worries, often seems worried | ○ | ○ | ○ |
| Helpful if someone is hurt, upset or feeling ill | ○ | ○ | ○ |
| Constantly fidgeting or squirming | ○ | ○ | ○ |
| Has at least one good friend | ○ | ○ | ○ |
| Often fights with other children or bullies them | ○ | ○ | ○ |
| Often unhappy, down-hearted or tearful | ○ | ○ | ○ |
| Generally liked by other children | ○ | ○ | ○ |
| Easily distracted, concentration wanders | ○ | ○ | ○ |
| Nervous or clingy in new situations, easily loses confidence | ○ | ○ | ○ |
| Kind to younger children | ○ | ○ | ○ |
| Often lies or cheats | ○ | ○ | ○ |
| Picked on or bullied by other children | ○ | ○ | ○ |
| Often volunteers to help others (parents, teachers, other children) | ○ | ○ | ○ |
| Thinks things out before acting | ○ | ○ | ○ |
| Steals from home, school or elsewhere | ○ | ○ | ○ |
| Gets on better with adults than with other children | ○ | ○ | ○ |
| Many fears, easily scared | ○ | ○ | ○ |
| Sees tasks through to the end, good attention span | ○ | ○ | ○ |

| D2. Overall, compared to other children of the same age, do you think that your child has difficulties in one or more of the following areas: emotions, concentration, behaviour or being able to get on with other people? | |
| --- | --- |
| No (Go to Part E) ○ | Yes, moderate difficulties ○ |
| Yes, minor difficulties ○ | Yes, severe difficulties ○ |

If you have answered "Yes", please answer the following questions about these difficulties:

| D3. How long have these difficulties been present? | |
| --- | --- |
| Less than a month ○ | ○ 6-12 months |
| 1-5 months ○ | ○ Over a year |

| D4. Do the difficulties upset or distress your child? | |
| --- | --- |
| Not at all | ○ |
| Only a little | ○ |
| Quite a lot | ○ |
| A great deal | ○ |

| D5. Do the difficulties interfere with your child's everyday life in the following areas? | | | | | |
| --- | --- | --- | --- | --- | --- |
|  | Not at all | Only a little | | Quite a lot | A great  deal |
| Home life | ○ | | ○ | ○ | ○ |
| Friendships | ○ | | ○ | ○ | ○ |
| Classroom learning | ○ | | ○ | ○ | ○ |
| Leisure activities | ○ | | ○ | ○ | ○ |

| D6. Do the difficulties put a burden on you or the family as a whole? | |
| --- | --- |
| Not at all | ○ |
| Only a little | ○ |
| Quite a lot | ○ |
| A great deal | ○ |

**PART E. FAMILY LIFE AND IMPACT OF THE CHILD’S ILLNESS**

| E1. In the last year, have any of the following happened to you? (Tick all that apply) | Yes | No |
| --- | --- | --- |
| Pregnancy problems | ○ | ○ |
| Separation or divorce | ○ | ○ |
| Marital problems | ○ | ○ |
| A close family friend or another relative (e.g. aunt, cousin, grandparent) died | ○ | ○ |
| Problems with your children | ○ | ○ |
| Your own job loss (not voluntary) | ○ | ○ |
| Your own job loss (not voluntary) | ○ | ○ |
| Money problems | ○ | ○ |
| Insufficient food | ○ | ○ |
| Other (please describe)…………………………………………................................................................. | | |

| E2. I am going to read some statements that people have made about living with an ill child. For each statement, please indicate at the present time if you would strongly agree, agree, disagree or strongly disagree (Please circle appropriate numbers) | | | | |
| --- | --- | --- | --- | --- |
| Strongly agree | 1 | | | |
| Agree | 2 | | | |
| Disagree | 3 | | | |
| Strongly disagree | 4 | | | |
|  | 1 | 2 | 3 | 4 |
| The illness is causing financial problems for the family | 1 | 2 | 3 | 4 |
| Time is lost from work because of hospital appointments | 1 | 2 | 3 | 4 |
| I am cutting down hours I work to care for my child | 1 | 2 | 3 | 4 |
| Additional income is needed in order to cover medical expenses | 1 | 2 | 3 | 4 |
| I stopped working because of my child’s illness | 1 | 2 | 3 | 4 |
| Because of the illness we are not able to travel out of the city | 1 | 2 | 3 | 4 |
| People in the neighbourhood treat us specially because of the my child’s illness | 1 | 2 | 3 | 4 |
| we have got little desire to go out because of my child’s illness | 1 | 2 | 3 | 4 |
| It is hard to find a reliable person to take care of my child | 1 | 2 | 3 | 4 |
| sometimes we have to change plans about going out at the last minute because of my child’s state | 1 | 2 | 3 | 4 |
| We see family and friends less because of the illness | 1 | 2 | 3 | 4 |
| Because of what we have shared we are a closer family | 1 | 2 | 3 | 4 |
| Sometimes I wonder whether my child will be treated “specially” or the same as a normal child | 1 | 2 | 3 | 4 |
| My relatives have been understanding and helpful with my child | 1 | 2 | 3 | 4 |
| I think about not having more children because of the illness | 1 | 2 | 3 | 4 |
| My partner and I discuss my child’s problems together | 1 | 2 | 3 | 4 |
| we try to treat my child as if he/she were a normal child | 1 | 2 | 3 | 4 |
| I don’t have much time left over for other family members after caring for my child | 1 | 2 | 3 | 4 |
| Relatives interfere and think they know what’s best for my child | 1 | 2 | 3 | 4 |
| Our family gives up things because of my child’s illness | 1 | 2 | 3 | 4 |
| Fatigue is a problem for me because of my child’s illness | 1 | 2 | 3 | 4 |
| I live from day to day and I don’t plan for the future | 1 | 2 | 3 | 4 |
| Nobody understands the burden I carry | 1 | 2 | 3 | 4 |
| Travelling to the hospital is a strain on me | 1 | 2 | 3 | 4 |
| Learning to manage my child’s illness has made me feel better about myself | 1 | 2 | 3 | 4 |
| I worry about what will happen to my child, in the future | 1 | 2 | 3 | 4 |
| Sometimes I feel like we live on a roller coaster: in crisis when my child is acutely ill, OK when things are stable | 1 | 2 | 3 | 4 |
| It is hard to give much attention to the other children because of the needs of my child | 1 | 2 | 3 | 4 |
| having a child with an illness makes me worry about my other children’s healthy | 1 | 2 | 3 | 4 |

**PART F: FAMILY SUPPORT**

| F3. This scale includes different types of assistance that people sometime find helpful. This questionnaire asks you to indicate how much you need help in each of these areas. Please circle the response that best describes your needs. Please answer all questions. | | | | | |
| --- | --- | --- | --- | --- | --- |
| To what extent do you feel a need for any of the following types of help or assistance | Never | Once in a while | Some  times | Often | Quite often |
| Someone to talk to about things that worry you | 1 | 2 | 3 | 4 | 5 |
| Someone to provide money for food, clothes and other things | 1 | 2 | 3 | 4 | 5 |
| Someone to care for your child on a regular basis | 1 | 2 | 3 | 4 | 5 |
| Someone to talk to about problems with raising your child | 1 | 2 | 3 | 4 | 5 |
| Someone to help you get services for your child | 1 | 2 | 3 | 4 | 5 |
| Someone to encourage you when you are down | 1 | 2 | 3 | 4 | 5 |
| Someone to fix things around the house | 1 | 2 | 3 | 4 | 5 |
| Someone to talk to who have similar experience | 1 | 2 | 3 | 4 | 5 |
| Someone to do things with your child | 1 | 2 | 3 | 4 | 5 |
| Someone to whom you can depend | 1 | 2 | 3 | 4 | 5 |
| Someone to hassle with agencies or businesses when I can’t | 1 | 2 | 3 | 4 | 5 |
| Someone to lend you money | 1 | 2 | 3 | 4 | 5 |
| Someone who accepts your child regardless of how he or she acts | 1 | 2 | 3 | 4 | 5 |
| Someone to relax and joke with | 1 | 2 | 3 | 4 | 5 |
| Someone to help with household chores | 1 | 2 | 3 | 4 | 5 |
| Someone to keep you going when things seem hard | 1 | 2 | 3 | 4 | 5 |
| Someone to care for your child in emergencies | 1 | 2 | 3 | 4 | 5 |
| Someone to talk to when you need advice | 1 | 2 | 3 | 4 | 5 |
| Someone to provide you and your children transportation | 1 | 2 | 3 | 4 | 5 |
| Someone who tells you about services for your child or family | 1 | 2 | 3 | 4 | 5 |

End of questionnaire.

Thanks you for your participation in the study.
